# EUS-FNA for a Pancreatic Neuroendocrine Tumor in a Four-Year-Old Daughter of a Woman Exposed to Radiation at Chernobyl

**DOI:** 10.1155/2012/462139

**Published:** 2012-06-17

**Authors:** Jesse Lachter, Marc S. Arkovitz, Sergey Postovski, Julian M. Waldner, Ron Shaoul, Offir Ben Ishay, Yoram Kluger

**Affiliations:** ^1^Department of Gastroenterology, The Ruth and Bruce Rappaport Faculty of Medicine, Technion-Israel Institute of Technology, Rambam Health Care Campus, Haifa 31096, Israel; ^2^Department of Pediatric Surgery, Meyer Children's Hospital, Rambam Health Care Campus, Haifa 31096, Israel; ^3^Department of Pediatric Oncology, Meyer Children's Hospital, Rambam Health Care Campus, Haifa 31096, Israel; ^4^Charité-Universitätsmedizin Berlin, Berlin, Germany; ^5^Department of Surgery, The Ruth and Bruce Rappaport Faculty of Medicine, Technion-Israel Institute of Technology, Rambam Health Care Campus, Haifa 31096, Israel

## Abstract

Pancreatic neoplasms in children are rare. Herein is reported the case of a four-year-old girl whose mother was exposed to radiation at Chernobyl that presented with obstructive jaundice and a mass suspected on CT and diagnosed by endoscopic ultrasound (EUS) with fine needle aspiration (FNA). This child is probably the youngest case of application of linear EUS with biopsy to be described. The diagnosis, management, and followup of children with this rare tumor are discussed.

## 1. Introduction

Pancreatic tumors in childhood are rare. Most specialists see few during their career. These tumors range in spectrum from benign duplication cysts, to primary pancreatic tumors to rare metastatic lesions, and primary lesions include the rare lymphoma or neuroendocrine tumors [1]. The presurgical workup for such patients is critical for ensuring optimal outcomes. Herein is reported a case of a four-year-old girl who presented with obstructive jaundice. EUS-guided FNA diagnosed a neuroendocrine tumor in the head of the pancreas with lymph node metastases.

## 2. Case Presentation

A four-year-old previously healthy girl presented with two months of abdominal pain and ten days of jaundice and acholic stools. There was no recent weight loss, no nausea or vomiting, fever, or change in mental status. There was no history of recent travel or trauma. There is no family history of pancreatic tumors in the past. The patients' mother had been exposed to radiation from Chernobyl in the former Soviet Union in 1989 and suffers from an unknown thyroid ailment. The mother takes no medication for her thyroid and has not had any surgery for a thyroid disorder.

On physical examination the child was jaundiced and had a palpable mass in the right upper quadrant that was nontender. The rest of her physical exam was unremarkable. Her laboratory results were remarkable for elevated direct bilirubin, LDH, GGTP, and alkaline phosphatase. Her alpha-fetoprotein, CEA and CA-125, were normal, her CA19-9 was elevated (see Table 1). All of her blood counts were within normal limits as were her coagulation tests. Fasting blood glucose analysis was normal on multiple tests.

**Figure 1:**
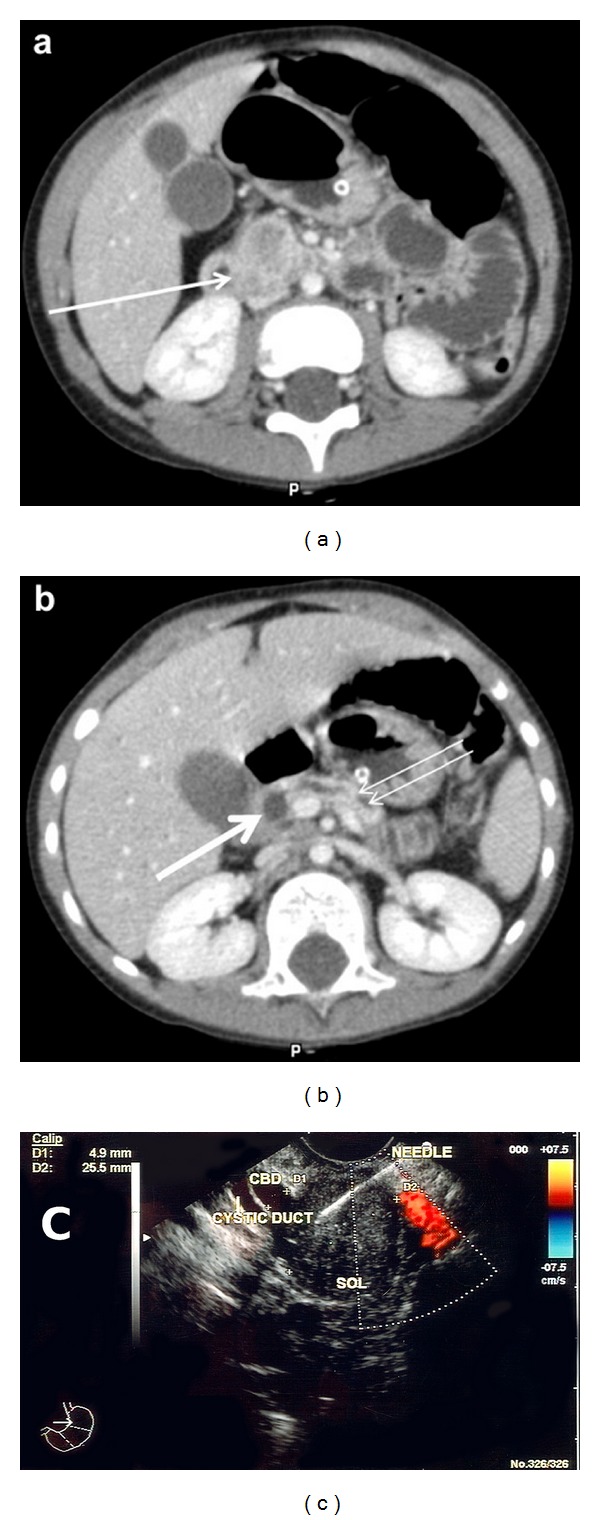
(a) CT Scan of Abdomen: Mass (Arrow) in head of pancreas. (b) CT Scan of Abdomen: Dilated Common (large arrow) and Pancreatic (Double arrow) ducts. (c) Endoscopic ultrasound image of lesion in head of pancreas.

 A CT scan revealed a 3 cm suspicious area in the head of the pancreas with dilatation of both the pancreatic and common bile ducts (Figures 1(a) and 1(b)). The gallbladder was markedly distended as were the intra-hepatic biliary radicles. A CT of the chest was negative/normal. Endoscopic ultrasound (PentaxFG38) revealed a well-circumscribed 3 cm lesion. FNA biopsy was performed (Figure 1(c)), and the results were consistent with a low-grade malignant epithelial tumor, most likely a nonfunctioning pancreatic neuroendocrine tumor, based on positive synaptophysin and chromogranin stains.

At laparotomy a large tumor in the head of the pancreas was palpated. In addition, several peripancreatic lymph nodes appeared grossly enlarged. A nonpylorus sparing Whipple procedure was performed including removal of the peripancreatic lymph nodes. The final pathology of the sample was a well-differentiated, non-functioning neuroendocrine carcinoma with 3 out of 12 lymph nodes sampled positive for tumor. There was no extension into the surrounding tissues or duodenum.

The patient had an uneventful postoperative recovery, tolerated a regular diet on postoperative day 5, and was discharged on the 9th postoperative day.

Pediatric oncologic consultation chose the “wait and see” approach, with no postoperative adjuvant therapy being administered. 18 months later, the child is apparently healthy, growing well, and with no current signs of disease.

## 3. Discussion

Primary pancreatic tumors in childhood are rare. Two of the largest series reported 17 and 25 patients each over the course of 30 years. In all series reported to date, the majority of these tumors are solid pseudopapillary tumors or pancreatoblastomas. Pancreatic NETs comprise a small, less than 20%, subset of these cases [2, 3].

Pancreatic neuroendocrine tumors are a subset of gastroenteropancreatic neuroendocrine tumors and can be broadly divided into functioning and non-functioning tumors based on their physiologic activity. In addition, the WHO divides these tumors into three broad categories based on tumor differentiation: well differentiated neuroendocrine tumor, well-differentiated neuroendocrine carcinoma, and poorly-differentiated endocrine carcinoma. Functioning tumors can arise from any of the neuroendocrine system. These tumors are usually sporadic but can be associated with genetic syndromes such as MEN-1, von Hippel-Lindau, neurofibromatosis 1, and tuberous sclerosis. Whatever the etiology, pancreatic neuroendocrine tumors are rare and are reported to be less than 3% of all primary pancreatic neoplasms [4].

The case presented here is unique for several reasons. The first is the age of the child. Of all the pancreatic neuroendocrine tumors reported in children, only one other has been as young as the child described here [2]. In addition, the mother of our patient reported an exposure to radiation from the Chernobyl reactor. Though it is impossible to know for sure if there is a correlation between the mothers “exposure and her child's [sic]” tumor, it is an interesting association, especially in such a young child.

Radiation exposure, such as the accident at Chernobyl, has led to increases in certain cancers, most notably thyroid cancer in children who were exposed and offspring of parents who were exposed. Elevated levels of Cs-137 have been documented in the endocrine glands of children exposed at Chernobyl particularly the pancreas, thyroid, and adrenals. In autopsy specimens examined children had significantly higher levels than adults who lived in the same geographic area of Belarus during the same time period of exposure [5]. Moreover, genetic mutations have been described in the offspring of people exposed to radiation at Chernobyl that were not present in offspring born before the exposure and that are not present in control children of parents not exposed at Chernobyl [6]. There has, however, been no demonstrated association between radiation exposure at Chernobyl and pancreatic neoplasms.

Endoscopic ultrasound-(EUS) guided biopsy is a well-described technique in the workup of pancreatic lesions in adults and has also been described in children. Previous reports have described the use of EUS in children to distinguish a true pancreatic neoplasm from jaundice due to pancreatitis in the head of the pancreas [7]. Most of these cases are in older children and adolescents. No published report was found of using the linear FG3813 mm echoendoscope used for taking FNA samples in such a young child. General anesthesia was performed by an anesthesiologist, without endotracheal intubation, so as not to make intubation of the large scope any harder. EUS demonstrated a well circumscribed mass in the head of the pancreas (Figure 1(c)) which was suggestive of a true neoplasm and not an inflammatory process. The nearly isoechoic pattern was most typical for a neuroendocrine tumor, the imaging unable to differentiate a neuroendocrine tumor from a neuroendocrine carcinoma. The common bile duct was dilated, no signs of vascular invasion or of liver or other distal metastasis were identified. The wide CBD seen in the figure takes into account the small size of the patient. The FNA-transduodenal biopsy (EUSN3needle, Wilson-Cook Corporation) was important in differentiating this primary pancreatic lesion from any metastatic lesion. Cytology specimens stained strongly positive for both synaptophysin and chromogranin, definitively making the diagnosis of neuroendocrine tumor. The only elevated “tumor marker” was CA 19-9, which is often a nonspecific marker of biliary obstruction that can be elevated to the levels seen in the present case in malignant as well as non-malignant processes, of the pancreas and of other organs [8].

The treatment and followup of non-functioning pancreatic neuroendocrine tumors is poorly defined. Most treatment and outcome studies of non-functioning pancreatic neuroendocrine tumors are in adults. In these studies, the long-term survival for a well-differentiated non-functioning carcinoma, such as in this child here, is approximately 50% at five-year followup. Despite these statistics, there is little to offer these patients in terms of adjuvant chemotherapy, or radiation and surgery with curative intent remains the treatment of choice. Resection of liver metastasis is the recommended treatment for patients with multiple liver metastases. Somatostatin analogs as well as streptozotocin and 5-FU have been recommended in cases where tumor has recurred and is deemed nonresectable. These patients can be followed with periodic ultrasounds as most tumor recurrence is in the liver.

Other studies such as PET scans and isotope-linked octreotide scans have been described but only on an experimental basis [4].

Pancreatic neuroendocrine tumors remain a rare primary pancreatic neoplasm described mostly in adults. Diagnostic possibilities considered included lymphoma, nonsecreting neuroendocrine tumor, solid and cystic pseudopapillary tumor, acinar cell tumor, autoimmune pancreatitis, and pancreatoblastoma. Pancreatic neuroendocrine tumors are rare in children but appear in about 5% of primary pancreatic neoplasms described in adults [9]. Treatment options for this tumor are limited, and it is best treated with surgical resection. Followup is clinical, other than the possible addition of serum chromogranin a, a marker of neuroendocrine tumors, which has been negative in this patient [10]. Herein is described a four-year-old girl, one of the youngest presented to date, who is the daughter of a woman exposed to radiation at Chernobyl. The diagnosis was established by EUS and biopsy. This child is probably the youngest case of application of linear EUS with biopsy to be described. A major goal of this report was to inform others that the large scope can be used in such small children successfully to establish clinically important diagnoses. While this report advocates for an innovative use of the EUS and FNA for a very small child, this case perhaps would not be considered appropriate for performance by a fellow, despite the very good results found by trainees in the recent report by Cotéetal. [11] on the training and success rates of EUS-guided FNA amongst experienced versus in-training echoendoscopists.

In followup a small child presented with a tumor metastatic to a few of the regional lymph nodes which were excised. Now, 18 months later, with no further medical care other than clinical followup, the patient is thriving and well.

## Figures and Tables

**Table 1:** Initial blood test values (units/liter and gram/liter), 1 KU/L means 10^3^ units/liter, bold values are out of range.

Test	Value	Normal range
Serum amylase	**20 U/L**	30–140** **U/L
Alkaline phosphatase	**1167 U/L**	50–160** **U/L
Lactate Dehydrogenase	**334 U/L**	60–225** **U/L
Gamma-glutamyl transferase	**1473 U/L**	50–60** **U/L
Alpha-fetoprotein	<2** **KU/L	0–8** **KU/L
CA-125	10.6** **KU/L	0–35** **KU/L
CA-19-9	**163 KU/L**	0–28** **KU/L
Bilirubin-direct	4.6 · 10^−2^ ** g/L**	<0.4·10^−2^ ** **g/L
CEA	0.6·10^−6^ ** **g/L	0–3·10^−6^ ** **g/L
